# Factors affecting the role of canonical Wnt inhibitor Dickkopf-1 in cancer progression

**DOI:** 10.3389/fonc.2023.1114822

**Published:** 2023-03-15

**Authors:** Dakota Doucet, Connor Brubaker, Donald Turner, Carl A. Gregory

**Affiliations:** ^1^ Medical Sciences Program, Texas A&M Health Science Center School of Medicine, Texas A&M University, Bryan, TX, United States; ^2^ Department of Statistics, Texas A&M University, College Station, TX, United States; ^3^ Department of Cell Biology and Genetics, Texas A&M Health Science Center School of Medicine, Texas A&M University, Bryan, TX, United States

**Keywords:** Dickkopf (DKK), Wnt signaling, stroma, cancer, immunomodulation, angiogenesis, survival, chemoresistance

## Abstract

**Background:**

The canonical Wnt inhibitor Dickkopf-1 (Dkk-1) has the capacity to modulate homeostasis between canonical and non-canonical Wnt pathways and also signal independently of Wnt. The specific effects of Dkk-1 activity on tumor physiology are therefore unpredictable with examples of Dkk-1 serving as either a driver or suppressor of malignancy. Given that Dkk-1 blockade may serve as a potential treatment for some types of cancer, we questioned whether it is possible to predict the role of Dkk-1 on tumor progression based on the tissue origin of the tumor.

**Methods:**

Original research articles that described Dkk-1 in terms a tumor suppressor or driver of cancer growth were identified. To determine the association between tumor developmental origin and the role of Dkk-1, a logistic regression was performed. The Cancer Genome Atlas database was interrogated for survival statistics based on tumor Dkk-1 expression.

**Results:**

We report that Dkk-1 is statistically more likely to serve as a suppressor in tumors arising from the ectoderm (*p* = 0.0198) or endoderm (*p* = 0.0334) but more likely to serve as a disease driver in tumors of mesodermal origin (*p* = 0.0155). Survival analyses indicated that in cases where Dkk-1 expression could be stratified, high Dkk-1 expression is usually associated with poor prognosis. This in part may be due to pro-tumorigenic role Dkk-1 plays on tumor cells but also through its influence on immunomodulatory and angiogenic processes in the tumor stroma.

**Conclusion:**

Dkk-1 has a context-specific dual role as a tumor suppressor or driver. Dkk-1 is significantly more likely to serve as a tumor suppressor in tumors arising from ectoderm and endoderm while the converse is true for mesodermal tumors. Patient survival data indicated high Dkk-1 expression is generally a poor prognostic indicator. These findings provide further support for the importance of Dkk-1 as a therapeutic cancer target in some cases.

## Introduction

The canonical Wnt/β-catenin (cWnt) pathway has garnered intense interest for its role in the regulation of cancer progression. The earliest work on cWnt signaling strongly implicated it as a driving factor in tumorigenesis based on initial observations that cWnt is upregulated by viral integration in murine breast tumors (1), and mutations of the adenomatous polyposis coli (APC) protein that constitutively drive cWnt signaling cause colon carcinoma (2, 3). Since this early work, a substantial body of literature has continued to bolster the understanding that cWnt signaling has the capacity to drive uncontrolled proliferation of tumor cells and support phenotypic adaptations that result in oncogenesis (4, 5).

To date, 19 homologous members of the Wnt ligand family have been discovered in human tissues (6), generally consisting of 350-400 amino acids and harboring posttranslational lipid and glycosyl modifications (6, 7). Wnt ligands can signal to the nucleus through at least 4 classes of pathway; the canonical or β-catenin mediated pathway, and the non-canonical planar cell polarity (PCP), calcium-mediated, and receptor tyrosine kinase triggered pathways (7–13). Wnt signaling is regulated at the intracellular level by cross-talking signaling pathways (9, 14), inhibitory intracellular molecules such as axin and APC (14, 15), and also in the extracellular space by at least six forms of secreted inhibitors (16). Frizzled related proteins (FRP), klotho, and Wnt inhibitory factor (WIF) all act by sequestering Wnt and preventing the ligand from binding to frizzled receptors. This class of Wnt inhibitor has the theoretical capacity to inhibit both canonical and non-canonical (ncWnt) forms of Wnt signaling. On the other hand, sclerostin (SOST), Mesd and dickkopf-1, 2 and 4 all target the LRP5/6 receptor and are therefore predicted to specifically inhibit the cWnt pathway.

Dickkopf-1 (Dkk-1) is the most intensely studied of the cWnt inhibitors, initially identified as a factor with the capacity for induction of a second head when its mRNA was injected into xenopus embryos (17). Dkk-1 is 35-40 kDa secreted glycoprotein consisting of two cysteine rich domains. Detailed structural analysis of Dkk-1 complexed with LRP5/6 indicate that both cysteine rich domains have the potential to interact with LRP5/6 (18, 19) whereas specific residues on second cysteine rich domain target the co-receptor kremen triggering internalization of the Dkk-1, Kremen, LRP5/6 complex (20–22). As well as its well-established role as a cWnt inhibitor, Dkk-1 has also been reported to signal independently of β-catenin in ways that enhance or inhibit malignant characteristics (23–30). Therefore, while Dkk-1 can act as a tumor suppressor in some cases by direct inhibition of cWnt, the complications of Dkk-1 signaling make its specific effects on tumor physiology unpredictable. Indeed, there are a growing number of examples of Dkk-1 serving as a driver of tumor expansion and metastasis in the literature (31, 32), and reports that high Dkk-1 levels serve as a poor prognostic indicator in many forms of hard tissue sarcoma (33), bladder cancer (34), hepatocellular carcinoma (35), cervical cancer (36), small cell lung (37), prostate (38) and breast (39) cancers.

Given that Dkk-1 blockade may serve as a valuable adjunct treatment for some types of malignancy (28, 40, 41), we questioned whether it is possible to predict the potential role of Dkk-1 (and the effect of its blockade) on tumor progression based on the tissue origin of the tumor. By systematic literature review, we tested the hypothesis that the developmental origin (ectoderm, mesoderm, endoderm) of the parental cell type of a tumor dictates whether Dkk-1 is more likely to serve as a tumor suppressor or tumor driver.

## Methods

### Database search

**Figure 1:**
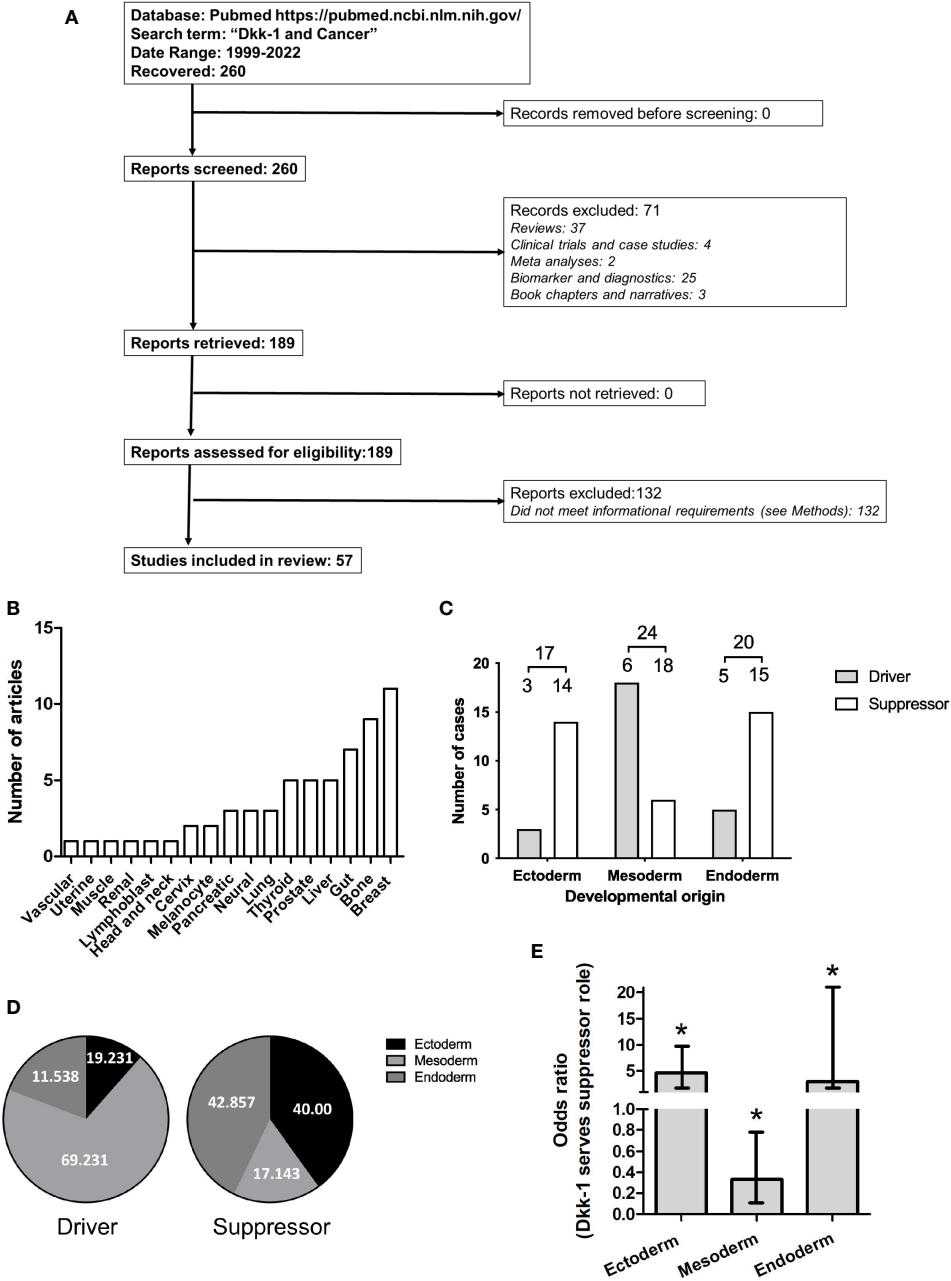
Dataset employed for systematic review of driver or suppressor status by Dkk-1 in tumors. **(A)**: PRISMA flow chart summarizing data curation. **(B)**: Frequency Tumor types covered by the dataset. **(C)**: Tumor types categorized based on developmental origin of the tumor and driver or suppressor status. Numbers are greater than the number of articles evaluated due to dual classification of some tumors. **(D)**: Distribution of data based on developmental origin of tumor and driver or suppressor status. **(E)**: Plot of odds ratios for Dkk-1 adopting a tumor suppressor role for each of the developmental layers. Error bars represent 95% confidence intervals and asterisk represents p < 0.05.

The Pubmed database (https://pubmed.ncbi.nlm.nih.gov/) was searched using the term “*Dkk-1 and cancer*” with the date range set to 1999-2022. After exclusion of reviews and editorials, original research articles that described Dkk-1 in terms a tumor suppressor or driver in a cancer cell or tumor growth were identified and shortlisted (**Figure 1A**). The developmental origin (ectoderm, mesoderm, endoderm) of each tumor or cancer cell in each study was categorized using definitions provided by the LifeMap Embryonic Development and stem Cell Compendium web resource (https://discovery.lifemapsc.com).

### Statistical analysis

Analysis was performed using R version 4.2.2 (2022–10–31). Studies were categorized based on whether Dkk-1 was reported as a driver or suppressor of tumorigenesis (functional categorization), then further categorized on the basis of tumor origin (ectoderm, mesoderm, endoderm). To determine the association between tissue layer and the role of Dkk-1, a logistic regression was performed. The independent and dependent variables were designated tissue layer and tumor suppression, respectively.

### Survival analysis

The DrBioRight bioinformatics platform https://drbioright.org was employed to interrogate The Cancer Genome Atlas (TCGA) database for overall survival statistics for patients harboring tumors with high or low Dkk-1 expression (high or low categorization was defined by Dkk-1 expression above or below the mean value). The dataset included specimens from brain, ovary, lung, prostate, uterus, bladder, testis, esophagus, pancreas, kidney, liver, cervix, soft tissue, breast, thymus, pleura, colon, stomach, bile duct, thyroid, head neck, bone marrow, rectum, skin, lymph nodes, adrenal gland, eye cancer patients. Survival data were analyzed by log rank test.

## Results

### Database search and categorization

The PubMed database was searched using the terms “*Dkk-1 and cancer*” between 1999-2022, yielding 260 results. Original research articles were shortlisted if they described Dkk-1 in terms of a tumor suppressor or driver in a cancer cell or tumor, and also specified the nature of the tumor or cancer cell studied, resulting in 57 remaining articles (**Tables 1** and **2**, **Figure 1A**). The studies were then sub-categorized, resulting in 22 studies describing Dkk-1 as a tumor driver (**Table 1**) and the remaining 35 describing Dkk-1 as a tumor suppressor (**Table 2**). The studies were then further categorized on the basis of tumor origin (ectoderm, mesoderm, endoderm), but in cases where tumors were indicated to be of mixed tissue origin or have underwent epithelial to mesenchymal transition (EMT), more than one developmental origin was assigned. Several tissues and all three developmental layers were broadly represented in the dataset (**Figures 1A, B**).

**Table 1:** List of studies where Dkk-1 is reported as a tumor driver.

PMID	Tissue (metastasis)	Layer	Secondary layer
35277659	Bone	Mesoderm	
30478297	Bone	Mesoderm	
28682874	Bone	Mesoderm	
27049730	Bone	Mesoderm	
24577091	Bone	Mesoderm	
21098705	Bone	Mesoderm	
35296660	Breast	Ectoderm	Mesoderm
24528599	Breast	Ectoderm	Mesoderm
26515701	Breast (bone)	Mesoderm	
25788273	Gut	Endoderm	
35269944	Liver	Endoderm	Mesoderm
27322059	Lung	Mesoderm	Endoderm
34884726	Muscle	Mesoderm	
33384994	Neural	Ectoderm	
27322059	Pancreas	Endoderm	
19711349	Pancreas	Endoderm	
18561248	Prostate (bone)	Mesoderm	
34437475	Prostate (bone)	Mesoderm	
20957670	Prostate (bone)	Mesoderm	
16140917	Prostate (bone)	Mesoderm	
35531363	Uterine	Mesoderm	
20847303	Vascular	Mesoderm	

**Table 2:** List of studies where Dkk-1 is a reported as a tumor suppressor.

PMID	Tissue	Layer	Secondary layer
24859848	Bone	Mesoderm	
20019092	Bone (Ewing)	Ectoderm	Mesoderm
35712490	Breast	Ectoderm	
30340507	Breast	Ectoderm	
27277008	Breast	Ectoderm	
25351982	Breast	Ectoderm	
20139903	Breast	Ectoderm	
18571836	Breast	Ectoderm	
18157634	Breast	Ectoderm	
18377964	Cervix	Mesoderm	
14555616	Cervix	Mesoderm	
35625973	Gut	Endoderm	
30655833	Gut	Endoderm	
22367735	Gut	Endoderm	
21317455	Gut	Endoderm	
18461655	Gut	Endoderm	
16491118	Gut	Endoderm	
19995224	Head/neck	Ectoderm	
29458569	Liver	Endoderm	
25344678	Liver	Endoderm	
17964517	Liver	Endoderm	
19746230	Liver	Endoderm	
15451431	Lung	Endoderm	
19148141	Lymphoblast	Mesoderm	
22420644	Melanocyte	Ectoderm	
17141200	Melanocyte	Ectoderm	
23354304	Neural	Ectoderm	
20920327	Neural	Ectoderm	
11840333	Neural	Ectoderm	
18632632	Prostate	Endoderm	Mesoderm
30405834	Renal	Mesoderm	
35198054	Thyroid	Endoderm	
24848709	Thyroid	Endoderm	
23261982	Thyroid	Endoderm	
22430125	Thyroid	Endoderm	

### Developmental origin and functional categorization of Dkk-1

Upon initial inspection of the data, the developmental origin of tumors where Dkk-1 is reported as a suppressor appeared to be predominantly ectodermal or endodermal (**Figures 1C, D**), whereas those tumors where Dkk-1 adopted a driver role appeared to be predominantly mesodermal in origin (**Figures 1C, D**). The logistic regression model confirmed this interpretation, with odds ratios obtained for tumor suppression of 3.00 (95% CI = [1.16, 9.22], *p* = 0.0334) for the endoderm layer, 0.33 (95% CI = [0.12, 0.79], *p* = 0.0198) for the mesoderm layer, and 4.66 (95% CI = [1.52, 20.24], *p* = 0.0155) for the ectoderm layer (**Figure 1E**). These odds ratios indicate that, for tumors originating in the mesoderm, there is an associated lower odds of observing a tumor suppressive role. Conversely, for tumors originating in the other two developmental layers, there is an associated higher odds of observing Dkk-1 serving a tumor suppressive role. Collectively, these data indicate that the developmental origin of the tumor can predict whether Dkk-1 adopts a driver or suppressor role.

### Dkk-1 expression and patient survival

**Figure 2:**
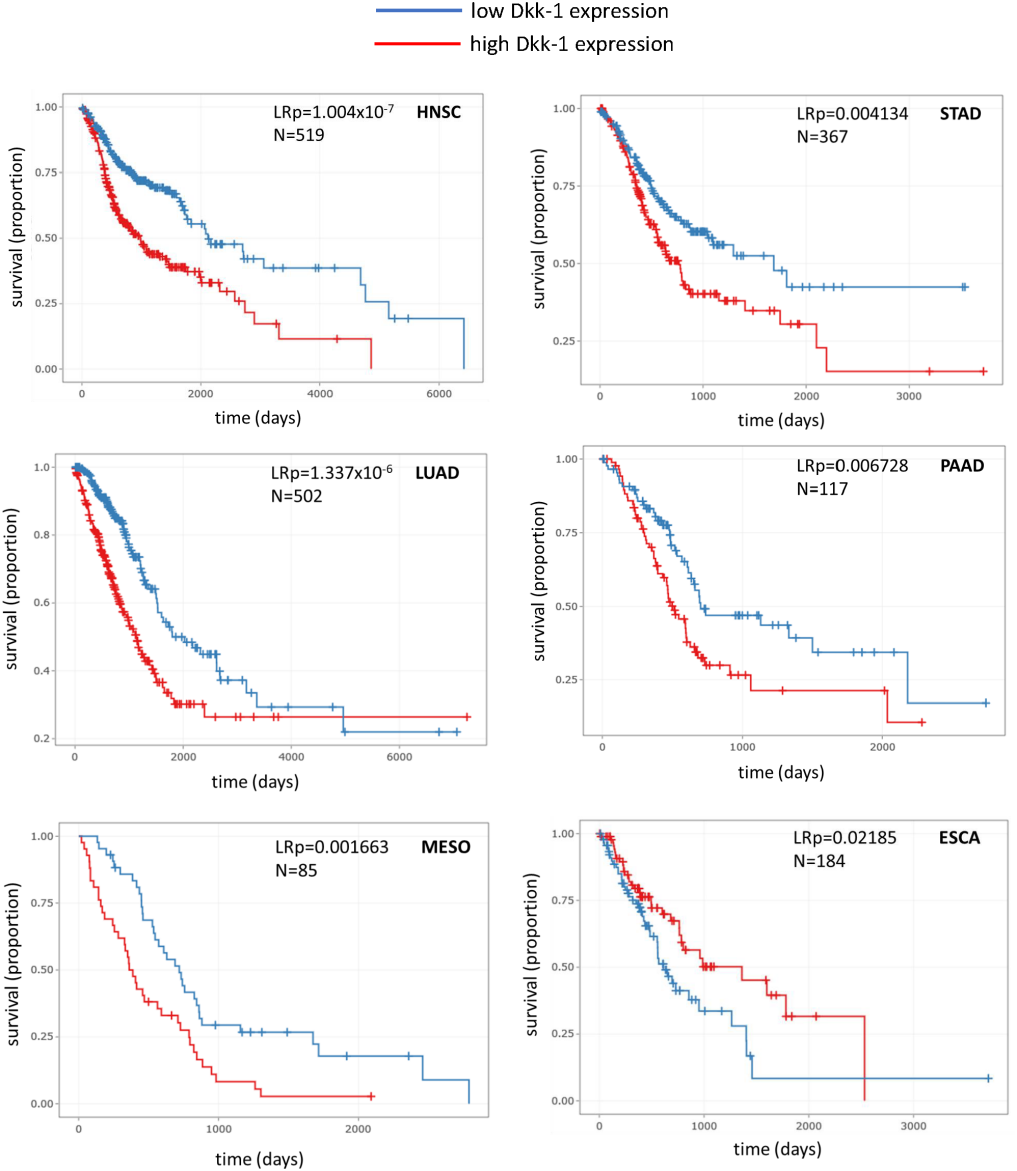
Kaplan-Meier survival plots for various tumor types where Dkk-1 has high or low transcriptional status (defined as above or below the mean value for the dataset respectively). HNSC, head and neck squamous cell carcinoma; LUAD, lung adenocarcinoma; MESO, mesothelioma; STAD, stomach adenocarcinoma; PAAD, pancreatic adenocarcinoma; ESCA, esophageal carcinoma. Data were recovered from TGCA and processed by the DrBioright platform. Data were analyzed by log-rank test.

The DrBioRight platform and the TCGA database was employed to correlate overall patient survival data with the level of Dkk-1 transcription in tumors. Tumor specimens with Dkk-1 transcription levels that were above the medium value was categorized as “*high expressors*” and those with values lower were categorized as “*low expressors*”. Of the six tumor subtypes where survival significantly differed with respect to Dkk-1 transcription, five had reduced survival probability associated with high Dkk-1 expression (lung adenocarcinoma, head and neck squamous cell carcinoma, mesothelioma, stomach adenocarcinoma and pancreatic adenocarcinoma) whereas one demonstrated a relatively weak association with survival and high Dkk-1 expression (**Figure 2**).

## Discussion

The data herein indicate that Dkk-1 plays multi-faceted and often contradictory roles in malignant disease progression. Dkk-1 is a unique member of the Wnt inhibitor family in that it can signal by Wnt-independent pathways, and in its capacity as a specific cWnt inhibitor, it can dysregulate the balance between canonical and ncWnt pathways, providing ample means for pleiotropic roles in different tissues.

As an inhibitor of cWnt, this apparently contradictory role for Dkk-1 probably arises in part from the multiple and diverse physiological roles of cWnt in tissue maintenance, where it serves as a proliferative inducer, survival factor, regulator of cell differentiation or pluripotency and modulator of attachment and migration (9, 10, 42–47). Intriguingly, the general mechanism of cWnt signal transduction is tightly conserved between tissue types and throughout evolution, raising the question of how cWnt maintains its remarkable diversity of function (47, 48). This likely arises from combinatorial activity of the seventeen extracellular Wnt ligands known to modulate cWnt/β-catenin-mediated signaling in human cells [The Wnt homepage: web.stanford.edu/group/nusselab/cgi-bin/wnt/human, KEGG: www.genome.jp/pathway/hsa04310] and ten subtypes of Frizzled receptor (7). Further levels of sophistication arise from crosstalk between cWnt and other pathways such as the SMAD pathway (49, 50), mitogen activated protein kinase pathways (50–52) and Hippo pathway components YAP and TAZ (53). The cell-specific role of cWnt, and subsequently Dkk-1, is therefore likely to be determined in large part by cell-inherent characteristics such as the expression profile of the Wnt ligands, Frizzled receptors, and the activity of cross-talking signaling pathways. It is also conceivable that stimuli from the microenvironment also plays a role in differentiating the output from cWnt signaling. This can occur through stimuli from adjacent stromal cells, most notably *via* β-catenin’s participation in adherens junction formation (54), or through attachment to extracellular matrix components (55).

This complexity is illustrated by the multiple roles cWnt plays in dorsoventral body axis specification and the formation of pre-dorsal mesenchyme of the Spemann-Mangold Organizer (56–58). This process, involving cytoskeletal organization, cell migration, differentiation and mitosis, is orchestrated predominantly by precise spaciotemporal control of cWnt signaling (48, 58). Similarly, cWnt plays multiple roles in cochleal development, orchestrating the proliferation, differentiation and dedifferentiation of epithelial and mesenchymal tissues (59). Due to lipid modifications that permit tethering to cell membranes, cWnt signaling is particularly adaptable to short range gradient-mediated signaling permitting localized cell proliferation and differentiation with high fidelity and at close quarters (7, 60). In contrast, the role of cWnt is far simpler in embryonic stem (ES) cells cultured on simple feeder layers, serving as an activator of downstream targets such as the cyclins and protooncogenes that drive proliferation (47).

In the adult, cWnt is also a potent proliferative inducer for several endodermal organs including liver (61–63), pancreas (64–66), and intestine (67, 68), and it plays a key role in the progression of malignancy that originates from these tissues (68–70). The predominant role of cWnt signaling in driving proliferation of intestinal cells is illustrated by the observation that abnormally high cWnt signaling results in accelerated cell division without profoundly affecting differentiation (68). In tissues of the ectoderm, cWnt also plays a predominantly mitogenic role for early neuroprogenitors (71, 72) but topologically and temporally regulated cWnt signaling is also necessary to orchestrate early stages of neurodifferentiation and morphogenesis (73–75). Canonical Wnt signaling plays a key role in driving proliferation of skin keratinocyte progenitors (76), and like neurons, it also plays an additional role in cell fate determination, with β-catenin deficient stem cells adopting an epidermal fate and β-catenin positive cells possessing the tendency to differentiate into follicular keratinocytes (77). In tissues of the mesoderm, cWnt adopts more extensive roles in lineage specification than in the other developmental layers. For example, in developing tendons of chicken limbs, downregulation of cWnt results in the differentiation of mesenchymal stem cells (MSCs) into tenocytes (78), whereas cWnt was necessary for differentiation of the same MSCs into osteoblasts (78–81). In hematopoietic stem cells (HSCs) self-renewal, plasticity and differentiation is determined by the amplitude of cWnt activity. Using APC mutants with varying capacity to perturb degradation of β-catenin, it was determined that slight elevation of cWnt activity was sufficient to upregulate *in vivo* engraftment and self-renewal of HSCs, but further elevation of cWnt activity enhanced differentiation into myeloid progenitor cells and T-cells in a dose-dependent manner (82). Cardiac development is regulated in part by cWnt signaling but this requires precise spaciotemporal exposure to stimuli (83, 84). For instance, activation of the cWnt pathway promotes differentiation of ES cells into cardiomyocytes but simultaneously suppresses differentiation into hematopoietic and vascular lineages. On the other hand, activation of cWnt signaling in the late phase of the embryoid body formation inhibits cardiomyocyte differentiation and promotes the expression of hematopoietic/vascular markers (83).

**Figure 3:**
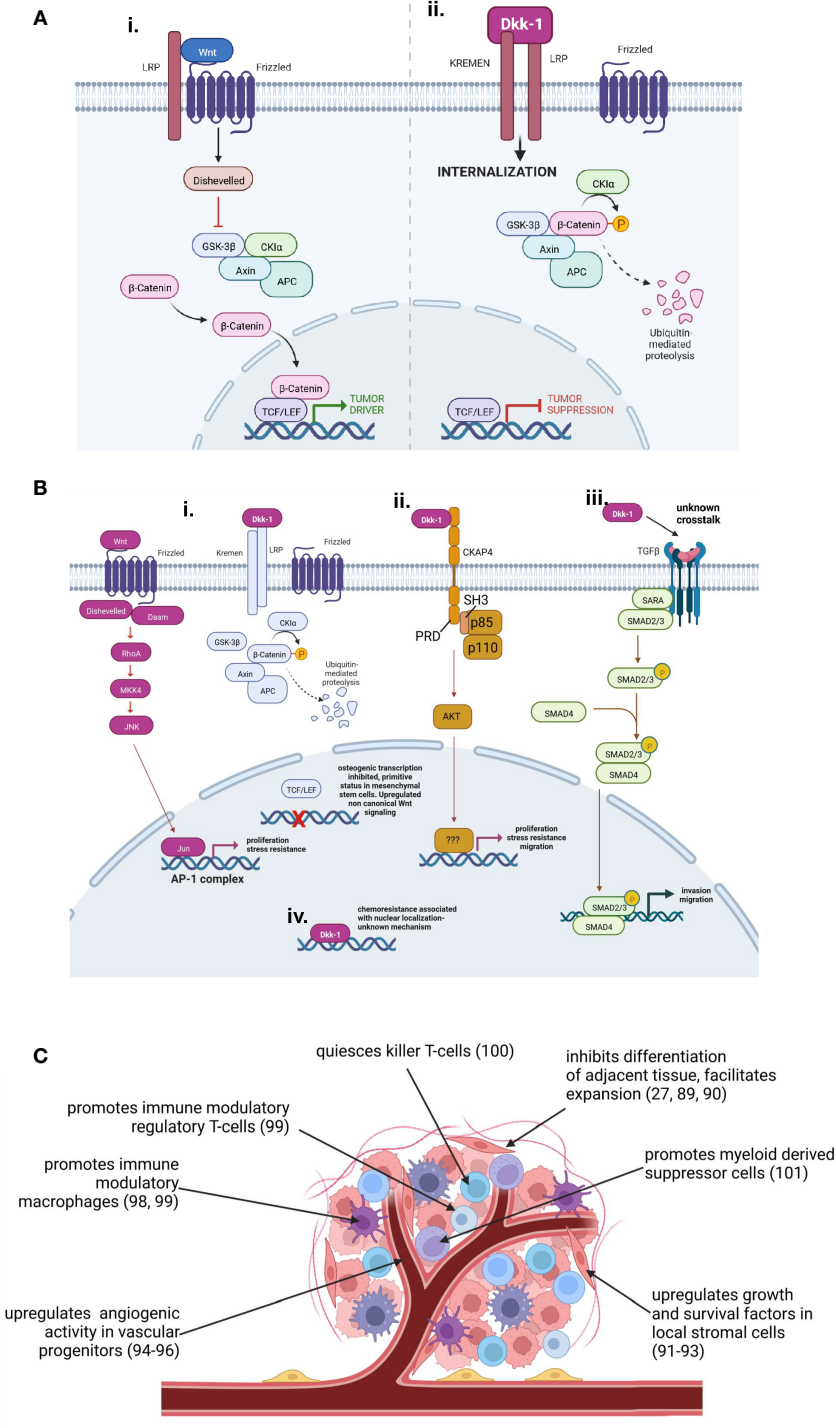
Tumor driver and suppressor roles adopted by Dkk-1. **(A)**: Dkk-1 acts as a tumor suppressor by inhibiting proliferative cWnt signaling. **(B)**: Tumor driver mechanisms of autocrine Dkk-1 activity. **(Bi)**: Dysregulation of balance between cWnt and ncWnt mechanisms in favor of ncWnt-mediated tumor survival pathways (e.g. ref: 27–30). Differentiation pathways mediated my cWnt may be affected, predisposing tumor cells to a more primitive, malignant phenotype (e.g. ref: 27, 85). **(Bii)**: Dkk-1 engages the CKAP4 receptor triggering PI3kinase and AKT activation resulting in enhanced proliferation, survival and migratory capacity (e.g. ref: 26, 86). **(Biii)**: Dkk-1 participates in synergistic crosstalk with TGFβ enhancing invasion and migratory capacity (e.g. ref: 14, 24). **(Biv)**: Dkk-1 interacts with nuclear components enhancing expression of survival factors that facilitate chemoresistance (e.g. ref: 87). **(C)**: Paracrine roles for Dkk-1 as a tumor driver modulating angiogenesis, immunoregulation, host tissue architecture and secretion of survival factors by stromal cells. Diagrams created by Biorender.com.

It is reasonable to posit that if cWnt has a predominantly proliferative role in the tissue origin of a tumor, the likelihood that Dkk-1 will serve as a tumor suppressor is high (e.g. in ectoderm and endoderm) (**Figure 3A**), whereas the role of Dkk-1 is more complex in tumors arising from tissues that tend to employ cWnt to direct cell lineage commitment (e.g. in mesoderm). In tissues where cWnt directs differentiation and lineage commitment, Dkk-1 can serve as a tumor driver by maintaining tumor cells in dedifferentiated state and facilitating their expansion. One of the best examples of this process is the reported derivation of malignant fibrous histiocytomas from MSCs by exposure to Dkk-1 (85). Dkk-1 can also modulate the balance between cWnt and ncWnt activity in favor of upregulated ncWnt signaling (**Figure 3Bi**). In some cases, upregulated ncWnt signaling caused by Dkk-1 enhances survival and proliferation of malignant cells through activation of stress resistance pathways often mediated by downstream JNK signaling (24, 27–30). Dkk-1 can also signal independently of cWnt/β-catenin in ways that enhance or inhibit malignant progression. For example, Dkk-1 has the capacity to activate the calmodulin dependent protein kinase II and so as to diminish tumor cell proliferation (23), interact with transforming growth factor beta (TGFβ) signaling so as to upregulate tumor invasion (24) (**Figure 3Biii**), and Dkk-1 can directly engage the cytoskeleton-associated protein 4 (CKAP4) receptor so as to trigger phosphatidylinositol-3-kinase/AKT and NF-κB signaling to increase cancer cell survival, motility and chemotherapeutic resistance (25, 26, 86) (**Figure 3Bii**). Dkk-1 has also been shown to localize to the nucleus in drug-resistant colorectal cancer, correlating with enhanced expression of survival factors such as aldehyde dehydrogenase. The mechanism of Dkk-1 is this case in not well understood, but direct or indirect association with chromatin is probable (87) (**Figure 3Biv**).

In tumors where expression data could be stratified into high and low Dkk-1 expression, patient survival data indicated high Dkk-1 expression was generally a poor prognostic indicator. With limited mechanistic data it is difficult to ascertain whether Dkk-1 is upregulated in these tumors in an effort to limit high cWnt signaling (88), or whether Dkk-1 is upregulated to serve a pro-tumorigenic role. The reason for this observation is probably partly attributed to the pro-tumorigenic role Dkk-1 can play in the progression of some malignancies (e.g. pancreatic ductal adenocarcinoma, osteosarcoma) but Dkk-1 also has the capacity to modulate the stroma of tumors (**Figure 3C**). In early studies of bone malignancies such as multiple myeloma, osteosarcoma and metastatic prostate cancer, it was found that Dkk-1 played a key role in bone engraftment (89) and local disruption of bone turnover to favor of osteolysis (27, 90). Dkk-1 was also found to irreversibly corrupt the ability of local osteoprogenitors to differentiate into osteoblasts, while forcing them to adopt a tumor-supportive role through provision of survival factors and osteolytic signals (91–93). Dkk-1 also has the capacity to act directly on local endothelial progenitors to stimulate angiogenesis (94–96). In more recent studies, Dkk-1 has also been shown to potently modulate the immunological landscape of the tumor in favor of immune evasion (97). In this capacity, Dkk-1 has been reported to stimulate the activity of immune suppressive macrophages in gastric cancer (98), increase numbers of regulatory macrophages and T-cells in cholangiocarcinoma (99), and Dkk-1 expression is correlated with natural killer T-cell quiescence in prostate cancer (100). Interestingly, Dkk-1 has been shown to enhance the activity of immune suppressive myeloid derived suppressor cells (MDSCs) by directly targeting cWnt activity, resulting in greater numbers of intratumoral MDSCs that can participate in immune evasion (101). Therefore Dkk-1, especially in tumors with a highly developed stroma, can act in both an autocrine and paracrine manner to drive tumor growth.

To our knowledge, this is the first time a relationship between the malignant driver/suppressor role of Dkk-1 and the developmental origin of the tumor has been demonstrated. While this preliminary work could offer a biological explanation for some of the controversies of the field, it has limitations. The hypothesis was tested using a relatively small data-set, limiting the current predictive power of this work, and another concern lies in the need to address paracrine actions of Dkk-1 on the tumor originating from host tissue. Further research utilizing the growing repository of clinically-annotated single-cell resolution transcriptomic data from the tumor and its associated stroma could provide substantial mechanistic clarification, and in the future, predict the efficacy of Dkk-1 blocking agents for a broad range of malignancies.

In summary, a systematic review of the literature indicated that Dkk-1 is more likely to play a suppressor role in tumors of ectodermal and endodermal origin where cWnt signaling is a predominantly proliferative force. In the case of mesodermal tissues, the role of cWnt is more complex, and Dkk-1 appears to be more likely to be associated with tumor promoting roles. Survival analyses using datasets from the TCGA database indicated that were where survival rates for patients can be stratified by high and low Dkk-1 expression, high Dkk-1 levels are usually associated with poor prognosis. This in part may be due to pro-tumorigenic roles Dkk-1 plays on tumor cells but also through immunomodulatory, developmental and angiogenic influences on the stroma too. Overall, the available data suggest that Dkk-1 blocking strategies may be effective in directly preventing the expansion of specific tumor types, but a more universal application for Dkk-1 blockade may lie in its capacity to target tumor-supporting components of the stroma.

## Data availability statement

The original contributions presented in the study are included in the article/supplementary material. Further inquiries can be directed to the corresponding author.

## Author contributions

DD and CG curated data, screened articles and wrote the manuscript. CB and DT performed the statistical analyses, CG conceptualized article, organized the work and acquired funding. All authors contributed to the article and approved the submitted version.
